# Ultrafast Time-Domain Spectroscopy Reveals Coherent Vibronic Couplings upon Electronic Excitation in Crystalline Organic Thin Films

**DOI:** 10.1021/acs.jpclett.4c02711

**Published:** 2024-10-31

**Authors:** Somayeh Souri, Daniel Timmer, Daniel C. Lünemann, Naby Hadilou, Katrin Winte, Antonietta De Sio, Martin Esmann, Franziska Curdt, Michael Winklhofer, Sebastian Anhäuser, Michele Guerrini, Ana M. Valencia, Caterina Cocchi, Gregor Witte, Christoph Lienau

**Affiliations:** † Institut für Physik, Carl von Ossietzky Universität, Carl-von-Ossietzky Str. 9-11, 26129 Oldenburg, Germany; ‡ Institut für Biologie, Carl von Ossietzky Universität, Carl-von-Ossietzky Str. 9-11, 26129 Oldenburg, Germany; § Center for Nanoscale Dynamics (CENAD), Carl von Ossietzky Universität, Carl-von-Ossietzky Str. 9-11, 26129 Oldenburg, Germany; ∥ Fachbereich Physik, Philipps-Universität Marburg, Renthof 7, 35032 Marburg, Germany; ⊥ Research Centre for Neurosensory Sciences, Carl von Ossietzky Universität, Carl-von-Ossietzky Str. 9-11, 26129 Oldenburg, Germany

## Abstract

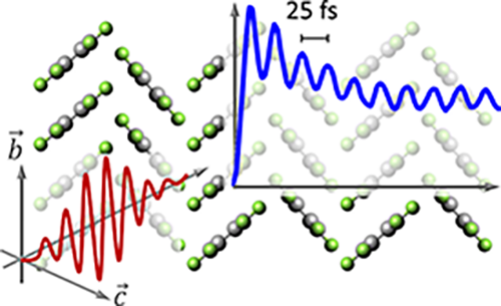

The coherent coupling between electronic excitations and vibrational modes of molecules largely affects the optical and charge transport properties of organic semiconductors and molecular solids. To analyze these couplings by means of ultrafast spectroscopy, highly ordered crystalline films with large domains are particularly suitable because the domains can be addressed individually, hence allowing azimuthal polarization-resolved measurements. Impressive examples of this are highly ordered crystalline thin films of perfluoropentacene (PFP) molecules, which adopt different molecular orientations on different alkali halide substrates. Here, we report polarization-resolved time-domain vibrational spectroscopy with 10 fs time resolution and Raman spectroscopy of crystalline PFP thin films grown on NaF(100) and KCl(100) substrates. Coherent oscillations in the time-resolved spectra reveal vibronic coupling to a high-frequency, 25 fs, in-plane deformation mode that is insensitive to the optical polarization, while the coupling to a lower-frequency, 85 fs, out-of-plane ring bending mode depends significantly on the crystalline and molecular orientation. Comparison with calculated Raman spectra of isolated PFP molecules in vacuo supports this interpretation and indicates a dominant role of solid-state effects in the vibronic properties of these materials. Our results represent a first step toward uncovering the role of anisotropic vibronic couplings for singlet fission processes in crystalline molecular thin films.

Organic semiconductor thin films have garnered significant attention in recent years due to their potential applications in advanced flexible optoelectronic devices such as organic photovoltaics, light-emitting diodes, and field-effect transistors.^1−3^ A crucial aspect of these materials is their anisotropic optical and charge transport properties, which arise from the shape anisotropy of the molecular entities causing an anisotropic molecular packing in the solid state.^4−6^ This anisotropy plays a vital role in determining the efficiency and performance of devices, as they directly influence the absorption of light, charge mobility, and overall electronic behavior of the organic films.

Other important characteristics of organic semiconductors are their particular pronounced vibronic couplings, which affect not only their energy landscape but also play an important role in their excited state dynamics and transport properties.^7−9^ While these characteristics have been investigated in detail,^4,5^ much less is known about the effect of the molecular alignment on the coupling between electrons and vibrational modes in organic solids, since the majority of experimental studies to date have been carried out on rather disordered samples. Here, organic single crystals or thin films with large crystalline domains offer the possibility to resolve these anisotropies by performing direction- and polarization-resolved measurements.^10^

In such thin films, the anisotropic molecular arrangement is expected to result in vibronic couplings that go beyond the common Condon approximation,^11−13^ in which the electronic transition dipole moment is independent of the nuclear coordinates and optical transition amplitudes are purely defined by the overlap of vibrational wave functions. Specifically, Herzberg–Teller couplings^14^ are expected if derivatives of the transition dipole moment along nuclear coordinates arise from nonadiabatic mixing of several electronically excited states,^13,15^ and conical intersections in the excited state potential energy surfaces may greatly influence the quantum dynamics after optical excitation.^16,17^ Emerging evidence indicates that such vibronic couplings may significantly affect the singlet fission processes in organic materials,^18−23^ where the role of vibronic couplings, and conical intersections in particular, are presently discussed.^24−29^

Perfluoropentacene (PFP) thin films, epitaxially grown on alkali halide substrates, are particularly interesting model systems to further extend the study of these topics.^10,30^ When grown on KCl(100) and NaF(100) substrates, they exhibit the same crystal structure with the characteristic herringbone packing motif but with different molecular orientations relative to the surface normal. In fact, the PFP molecules are standing almost upright on NaF(100), their long axis is oriented almost parallel to the substrate on KCl(100).^10,30^ On both substrates, the molecules form large, laterally extended domains. This structural peculiarity has enabled detailed directional polarization-resolved infrared vibrational spectroscopy and fission yield studies.^10,30^ Significant differences in polarization-dependent singlet fission yields have been observed in these crystals, suggesting that their anisotropic molecular packing affects singlet fission.^31^ Broadband pump–probe spectroscopy showed that the slip-stack molecular packing along the *b⃗*-axis of the crystals enhances singlet–triplet mixing and is favorable for singlet fission.^31^ The time resolution in these measurements,^31^ however, was not yet sufficient to resolve coherent vibrational motion. Therefore, little is known so far about the interplay between molecular packing and vibronic couplings in crystalline PFP thin films.

Effects of the molecular orientation on the infrared spectra of crystalline PFP films have been investigated in ref.,^30^ revealing vibrational Davydov splitting and collective mode polarizations as signatures of intermolecular couplings in the crystal. For the coupling to electronic excitations, however, Raman-active modes are most relevant. In case of PFP thin films, first results of Raman measurements have been obtained on highly oriented pyrolytic graphite.^32^ On graphite, however, PFP forms a π-stacked phase, different from the herringbone packing on alkali halides.^33^ Stronger vibronic couplings are expected for perfluorinated acenes than for nonfluorinated acenes, since the higher mass of fluorine than hydrogen enhances the reduced mass.^34^ Consequently, also the carbon atoms of the backbone have a much larger vibration amplitude, making it relevant to investigate vibronic couplings in PFP films in more depth.

Here, we use time-resolved vibrational spectroscopy with 10 fs time resolution and Raman spectroscopy to study vibronic couplings upon electronic excitation in crystalline PFP thin films grown on KCl(100) and NaF(100) substrates. Our results show that vibronic couplings to high-frequency carbon–carbon-stretching modes with 25 fs period induce large amplitude modulations of the nonlinear spectra. Polarization-dependent multimode interference patterns are signatures of the effect of molecular orientation on the vibrational motion which go beyond simplified single-molecule pictures for the optical response. Our results present a step forward in studying the effects of molecular order on vibronic couplings and singlet fission in molecular crystals.

We experimentally study the optical properties of single crystalline PFP thin films epitaxially grown on NaF(100) and KCl(100) substrates with thicknesses of 200 and 100 nm, respectively. These films were prepared under high vacuum conditions by molecular beam deposition and characterized in earlier work using several techniques including X-ray diffraction (see Figure S1 in the Supporting Information), atomic force microscopy (AFM), polarized optical microscopy, as well as infrared and UV/vis spectroscopy (see also Figure S2 in the Supporting Information).^10,30^ On both substrates, the PFP molecules crystallize in their bulk phase with a herringbone structure exhibiting a rectangular but slipped stacking of the two molecules in the unit cell^35^ (Figure. 1a,b). In the resulting triclinic lattice, the unit vector *a⃗* has a length of 15.5 Å and points approximately along the long axis of the PFP molecules. On the other hand, the *b⃗* vector (4.5 Å) is aligned along the slip-stacking direction while the *c⃗* vector (11.5 Å) points along the herringbone stacking direction.^30,35^ On NaF (Figure. 1a), the molecules stand almost upright on the substrate and *b⃗* coincides with the ⟨010⟩ azimuth direction of the substrate. On the other hand, on KCl (Figure. 1b), the molecules lie recumbently on the surface^10^ and their long axis encloses a small angle of ≃8° with respect to the substrate plane. Here, *b⃗* closely matches the ⟨011⟩ azimuth direction of the substrate while the *c⃗* vector points toward the substrate surface plane.

**Figure 1:**
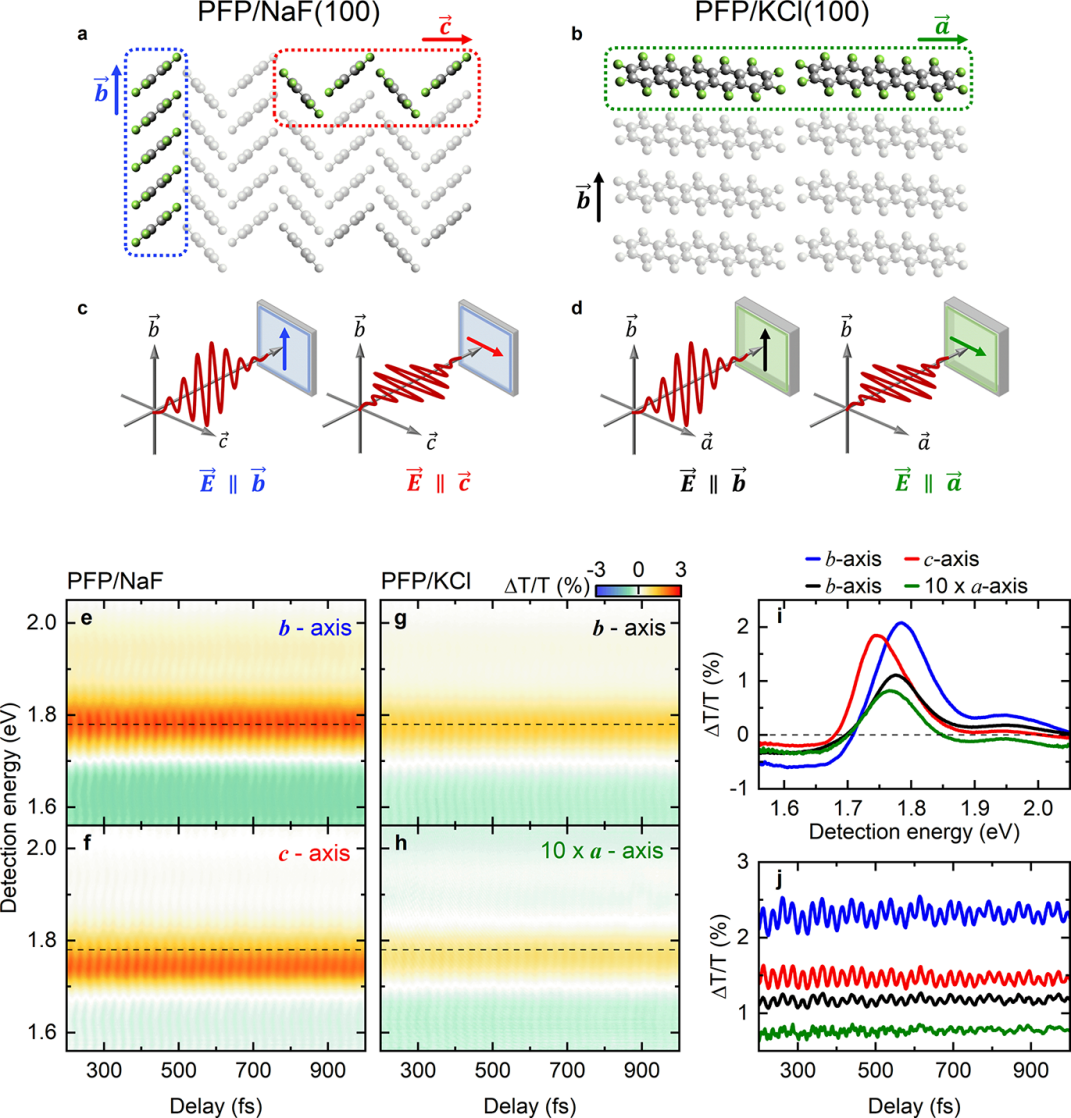
Correlation between crystalline orientation and optical response of perfluoropentacene (PFP). Molecular stacking patterns of (a) a 200 nm thick single-crystalline PFP film on a NaF(100) substrate and (b) a 100 nm thick single-crystalline PFP layer on a KCl(100) substrate. On NaF(100), with upright standing molecular orientation, the (*b⃗c⃗*)-plane of PFP is parallel to the substrate. For KCl, with recumbent molecular orientation, the (*a⃗b⃗*)-plane is parallel to the substrate. (c, d) Scheme of the polarization of the co-linearly polarized pump and probe lasers used to selectively excite and probe PFP on NaF (c) and PFP on KCl (d) with light polarized along one of the crystal axes. Differential transmission (Δ*T*/*T*) map recorded for linear polarization along the (e) *b⃗*- and (f) *c⃗*-axes of PFP on NaF and along the (g) *b⃗*- and (h) *a⃗*-axes of PFP on KCl. (i) Time-averaged Δ*T*/*T* spectra obtained from the Δ*T*/*T* maps in (e-h). The polarization of laser pulses along a specific crystal orientation are color-coded as in (e-h). (j) Cross sections of the Δ*T*/*T* maps in (e-h) as a function of delay time for a fixed probe energy, indicated by the dashed lines in (e-h). The Δ*T*/*T* for polarization along *a⃗*, magnified by a factor of 10 in (h-j) stems from a small number of residual minority domains oriented along *b⃗*.

AFM imaging and polarized microscopy experiments revealed the formation of extended, crystalline rotational domains with dimensions of several tens of microns,^10^ occurring in orthogonal domains due to the surface symmetry of the alkali halide surfaces. This film structure offers unique opportunities for performing linear and nonlinear optical experiments on single-crystalline domains, even if the spatial resolution does not reach the diffraction limit.^31^Figure. 1c,d schematically illustrate the incident polarization of the pump- and probe pulses in relation to the crystal axes of the films. On NaF(100), with the molecular long axis pointing nearly perpendicular to the substrate plane, linearly polarized light at normal incidence can excite the crystalline molecular film with the axis of polarization containing the *b⃗* or *c⃗* vectors, depending on the azimuthal polarization. On KCl(100), with recumbent molecular orientation, the incident electric field can be polarized along the *a⃗*- or *b⃗*- axes (see Figure S2 of the Supporting Information).^31^ Such an alignment of the incident polarization has a significant effect on the nonlinear optical response of the crystals. This is demonstrated in Figure. 1e-h showing the results of ultrafast pump–probe experiments. The targeted addressing of individual azimuthal domains is possible because the PFP films form laterally extended areas (<500 μm) with a preferred orientation. Polarization microscope images show that more than 90% of such areas exhibit only one azimuthal orientation with only small admixtures of the 90° rotation domains.

We use this special film geometry for time- and polarization-resolved pump–probe measurements, employing broadband pulses with a spectrum covering a range from 590 to 810 nm, i.e., 1.53 to 2.1 eV.^36^ Their 10 fs pulse duration (full width at half-maximum of their intensity profile, see Section 3 of the Supporting Information) is short enough to resolve vibronic couplings to all high-frequency vibrational modes of the thin films. The pulse spectrum fully overlaps the lowest-lying singlet exciton (X_S1_) of the PFP crystals at about 1.8 eV and all relevant vibrational sidebands (see Figure S2 in the Supporting Information). Collinearly polarized pump–probe measurements are performed at room temperature and under ambient conditions. The pulses are focused to a spot size of around 30 μm on the sample, sufficient to isolate oriented single-crystalline domains. The pump-induced change in probe transmission  is recorded as a function of pump–probe time delay *t*_*d*_ and detection energy *E*_*D*_. More details of the experimental setup are described in previous work (see also the Methods section).^36−40^ The fluence of pump and probe pulse is carefully controlled to ensure that all experiments are performed in the regime of third-order nonlinearities (Section 4 of the Supporting Information). Resulting pump–probe maps are displayed in Figure 1e-h for time delays between 200 fs and 1 ps.

For PFP on NaF(100), we first set the polarization of the pump and probe pulses parallel to the *b⃗*-axis of the film. To do this, we maximize a distinct excited state absorption (ESA) band that appears at energies below the singlet exciton resonance. This ESA is observed as a negative signal, Δ*T*/*T* < 0, in the range between 1.55 and 1.7 eV in Figure 1i (blue line), similar to earlier pump–probe measurements.^31^ The ESA has been assigned to an excimer state with its transition dipole moment oriented along the *b⃗*-axis.^31^ Here, we take this ESA as a marker that pump- and probe are oriented along *b⃗* as it vanishes almost completely when rotating the laser polarization by 90°, i.e., when exciting and probing the sample with light that is linearly polarized along *c⃗* (red curve in Figure 1i). Variation of the laser polarization confirms that the ESA is minimized for the setting shown in Figure 1f. This contrast in ESA ensures that a crystalline region with well-defined molecular orientation is excited and thereby allows for the identification of individual rotational domains in the PFP films. Not only the amplitude of the ESA but also the signature of X_S1_ in the pump–probe spectra changes significantly when rotating the laser polarization. All experiments show an enhanced transmission, Δ*T*/*T* > 0, near X_S1_. This reflects ground state bleaching (GSB) and stimulated emission (SE) from the singlet exciton. Importantly, the center of X_S1_ shifts spectrally from 1.78 eV for a polarization along *b⃗* (blue curve in Figure 1i) to 1.74 eV for a polarization along *c⃗* (red curve in Figure 1i). A similar spectral shift of 30 meV was observed in linear UV/vis spectra of PFP on NaF(100) between X_S1_ for excitations along *b⃗* and *c⃗* and assigned to an excitonic Davydov splitting in the molecular crystal (see Figure S2).^41^ PFP molecules with their optical transition dipole aligned along the short axis can coherently exchange energy with their nearest neighbors within and across the unit cell by near-field dipole–dipole coupling. In the strong coupling limit, this result in a delocalization of the electronic wave function across several molecules.^42^ The resulting Davydov components of the excitons are split in energy and have transition dipole moments oriented along the *b⃗*- and *c⃗*-axes, respectively. For PFP on NaF(100) both the energy splitting between the two states and the orthogonal orientation of the dipole moments are resolved in the pump–probe measurement (Figure 1e,f).

In contrast, for PFP on KCl(100), the pump and probe laser polarizations can be aligned along the *a⃗*- or *b⃗*-axes. For excitation along *b⃗*, the recorded pump–probe map (Figure 1g) is basically identical to that in Figure 1e (NaF substrate, *b⃗*-axis), except for an overall reduction in amplitude by a factor of 2 that reflects the decrease in film thickness from 200 nm on NaF(100) to 100 nm in KCl(100). For excitation along *a⃗*, the amplitude of the pump–probe signal largely reduced by more than one order of magnitude (note that the Δ*T*/*T* for excitation along *a⃗* in Figure 1h-j are magnified by a factor of 10. In this case, X_S1_ cannot be excited since its dipole moment is oriented perpendicular to the laser polarization. Polarization-resolved UV–vis spectra indicate^10^ (see Figure S2) that the lowest exciton resonance with dipole moment orientation along *a⃗* appears at 2.8 eV, i.e., it lies outside the spectral window of the pump laser. Consequently, the pump–probe signal shown in Figure 1h probes mainly residual excitations of X_S1_ that are oriented along *b⃗*. These signals therefore likely arise from a small admixture of 90° rotation domains with exciton transition dipole moments oriented along the *b⃗*-axis. The relative contribution of the low-energy ESA to the signal is as large as in Figure 1e.

All pump–probe transients show substantial oscillatory modulations on top of these incoherent signal contributions, which reflect pump-induced vibrational motion in the thin films. Representative signals are shown in Figure 1j for the detection energies marked by dashed lines in Figure 1e-h. To analyze these oscillations in more detail, we first compare transient pump–probe spectra for PFP on NaF(100) for excitations along *b⃗* (Figure 2a) and *c⃗* (Figure 2b), respectively. On these substrates, spurious cross-phase modulations induced by coherent light scattering contributions during and shortly after the pump–probe overlap are so weak that their amplitude is less than 10% of the desired pump–probe signal. This is verified via reference measurements on the bare substrate. The detection energies are selected to maximize the modulation contrast of the oscillations with a period of 25.3 fs (164 meV, 1320 cm^–1^) that dominate these signals. This oscillation period agrees very well with a mode period of 24.9 fs (166 meV, 1340 cm^–1^) estimated from the vibronic side peaks of UV/vis spectra (see Figure S2). The modulation contrast is taken as the ratio between the peak-to-peak oscillation amplitude on the Δ*T*/*T* transients and the cycle-average of Δ*T*/*T* and directly measures the strength of the vibronic coupling to the corresponding vibrational mode.^43^ For both polarizations, the data show a pronounced modulation with a period of 25 ± 1 fs. Specifically, for polarization along *c⃗* the modulation contrast is ∼30%. This demonstrates that the excitonic state is strongly coupled to a high-frequency mode of the PFP molecules at 1320 cm^–1^ and that optical excitation of the thin film induces coherent vibrational wavepacket motion along the corresponding nuclear coordinate.

**Figure 2:**
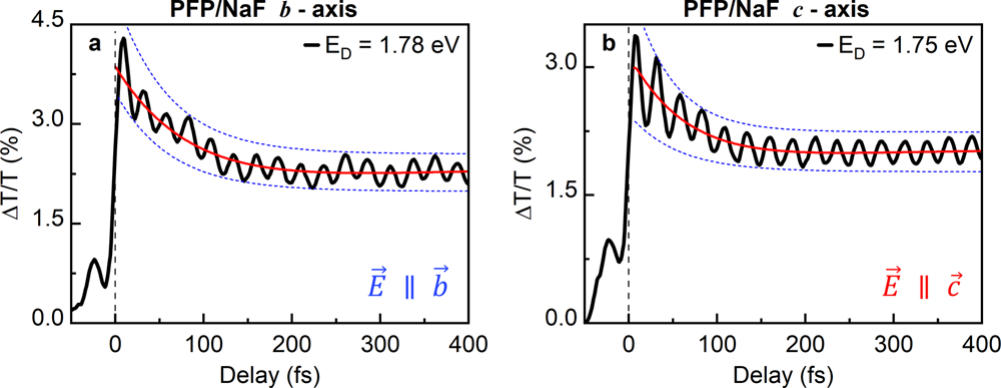
Transient differential transmission *ΔT*(*t*_*d*_,*E*_*D*_)/*T* recorded for PFP on NaF(100) with pulses polarized (a) along the *b⃗*-axis at a detection energy of 1.78 eV and (b) along the *c⃗*-axis at a detection energy of *E*_*D*_ = 1.75 eV. The polarization direction is illustrated in the insets. Both plots show a dominant modulation with 25 fs period although for polarization along *b⃗*, low-frequency oscillation beatings are more pronounced. The solid red and dashed blue lines are biexponential decay functions and are introduced to guide the eye.

In a simplistic displaced harmonic oscillator (DHO) model,^43^ in which the electronically excited (X_S1_) state couples to the vibrational mode of an individual harmonic oscillator, this modulation amplitude is expected for a dimensionless displacement of Δ ≈ 0.6 ± 0.1. This displacement corresponds to a Huang–Rhys factor *S* = Δ^2^/2 = 0.18, representing the average number of vibrational quanta involved in the electronic transition.^12,44^ For an excitation along the *b⃗*-axis, the pump–probe transient is still predominantly modulated with a 25 fs period. Here, however, the modulation amplitude is slightly reduced, and the high frequency modulation interferes with an additional lower-frequency mode with a period of 86 ± 2 fs (48 meV, 384 cm^–1^). This finding already suggests that the vibronic couplings in the thin films strongly depend on the polarization direction of the incident light pulses.

For a more quantitative assessment of these vibronic couplings, we calculate residual maps of the coherent oscillations at larger delays. To do so, we fit a multiexponential decay ⟨Δ*T*⟩(*t*_*d*_,*E*_*D*_) to the pump–probe transients for each *E*_*D*_ (see Section 5 of the Supporting Information) and subtract it from the data. This decay characterizes the incoherent contributions to the pump–probe transients. The resulting residual map R(*t*_*d*_,*E*_*D*_) = (Δ*T*(*t*_*d*_,*E*_*D*_) - ⟨Δ*T*⟩(*t*_*d*_,*E*_*D*_))/*T* is depicted in Figure 3a for PFP on NaF(100) excited with light polarized along *b⃗*. Pronounced oscillations in these residuals are observed in the probe energy range between 1.73 and 1.81 eV, close to the two Davydov components of the lowest energy singlet exciton (see Figure S2 in the Supporting Information). A distinct node in the modulation appears at 1.81 eV. The modulation amplitude is significantly larger for energies below 1.81 eV than for higher energies. Cross sections of the residuals in Figure 3b for energies of 1.78 eV (black) and 1.85 eV (red), chosen to maximize the oscillation amplitude on either side of the node, demonstrate this asymmetry. We emphasize that such a behavior cannot be explained based on a simple DHO model for which the analytical solutions of the pump–probe spectra are well-known.^43,45^ In the DHO model, the dependence of the modulation amplitude on *E*_*D*_ follows the first spectral derivative of the exciton resonance *d*(Δ*T*(*E*_*D*_)/*T*)/*dE*_*D*_. For a characteristic Lorentzian or Gaussian line shape of this resonance, a symmetric variation of the modulation amplitude is predicted by the model around the central node,^45^ in contrast to what is seen experimentally.

**Figure 3:**
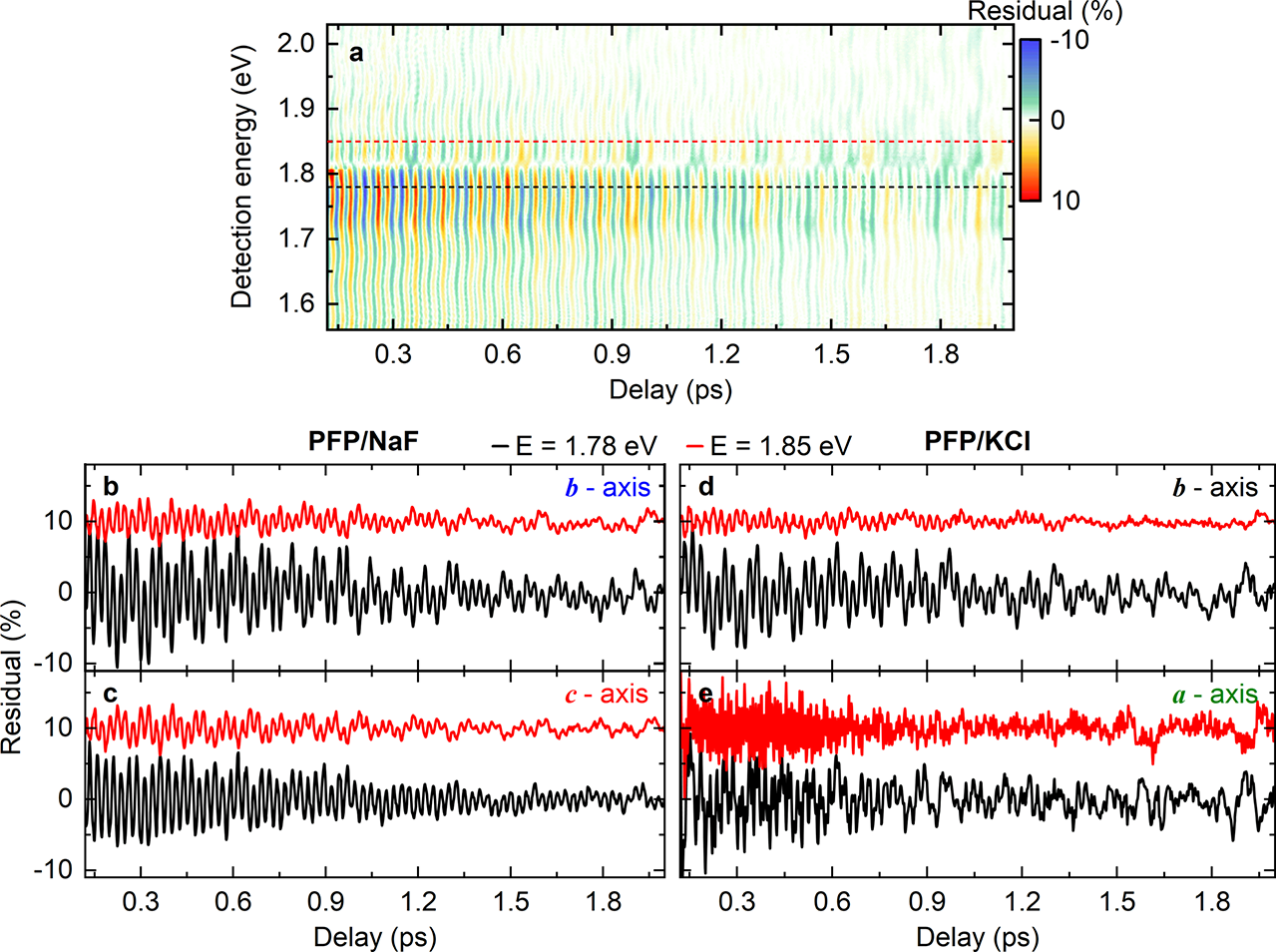
(a) Residual map showing the oscillatory modulation of the Δ*T*/*T* signals in Figure 1e, recorded for PFP on NaF for co-linearly polarized pump and probe pulses polarized along the *b⃗*-axis of PFP. In these residuals, slowly decaying incoherent Δ*T*/*T* signals have been subtracted from the pump–probe data to emphasize the oscillatory modulation induced by coherent lattice vibrations. The residuals are shown for waiting times between 0.12 and 2.0 ps. (b) Cross section through the residual map in (a) for two representative probe energies of 1.78 eV (black) and 1.85 eV (red), marked as dashed line in (a). The signals are dominated by a high-frequency oscillation at 25 fs and superimposed by several different beating patterns. (c-e) Cross sections through the residual maps for PFP on NaF (*c⃗*-axis) (c), PFP on KCl (*b⃗*-axis) (d) and PFP on KCl (*a⃗*-axis) (e). The cross sections are shown at probe energies of 1.78 eV (black) and 1.85 eV (red) and for time delays between 0.12 and 2.0 ps. In (b-e), the residuals at the two probe energies are vertically shifted by 10% for clarity.

For PFP on both substrates (Figure 3b-e) we find that the residuals are dominated by large amplitude 25 fs oscillations, regardless of the laser polarization. These oscillations persist for up to 6 ps, the maximum range of time delays chosen in our experiments. For excitation along *b⃗*, these oscillations are superimposed by lower-frequency oscillations with an 85 fs period. The same characteristic beating pattern is seen for *b⃗* excitation of PFP on both NaF and on KCl substrates (Figures 3b and 3d, black lines). Also, for excitation along *a⃗* on KCl(100) (Figure 3e), the same beating pattern but now much reduced in amplitude and superimposed by noise fluctuations. The amplitude is reduced since only a small admixture of 90° rotation domains with exciton transition dipole moments oriented along the *b⃗*-axis is excited.

A substantially different beating pattern arises when exciting and probing PFP on NaF(100) with polarization along *c⃗*. In this case (Figure 3c, black line), the residuals are largely dominated by the 25 fs oscillations and slower beating patterns are much weaker. Thus, for PFP on NaF(100), the oscillatory modulation of the pump–probe signal and, thus, the vibronic couplings, significantly depend on molecular orientation in the thin film. Such a pronounced anisotropy of the nonlinear response cannot be explained by a DHO model where a single electronic state is coupled to one or several harmonic oscillator modes. Instead, this response is a signature of anisotropic vibronic couplings in a molecular crystal in which delocalized, Davydov-split exciton resonances are coupled to vibrational modes in the crystalline solid.

To validate this conclusion, we present in Figure 4 the amplitude of the Fourier transforms of these residuals as a function of detection energy and mode frequency. For calculating the Fourier transform spectra, we used pump–probe transients for waiting times between 0.12 and 6 ps, except for the measurement performed on KCl with *a⃗*-axis excitation, where 3 ps scans were employed. Fourier transform spectra are calculated directly from the residuals, without applying any window functions. Maps of the amplitude of these spectra after integration along *E*_*D*_ are presented as black lines in Figure 5. These data are compared with polarization-resolved spontaneous Raman scattering measurements (red lines) performed on the same samples used for pump–probe spectroscopy. The Raman spectra were recorded with off-resonant excitation at 785 nm (1.58 eV). The spatial resolution in the measurement was ∼1 μm, ensuring excitation of single-crystalline domains.

**Figure 4:**
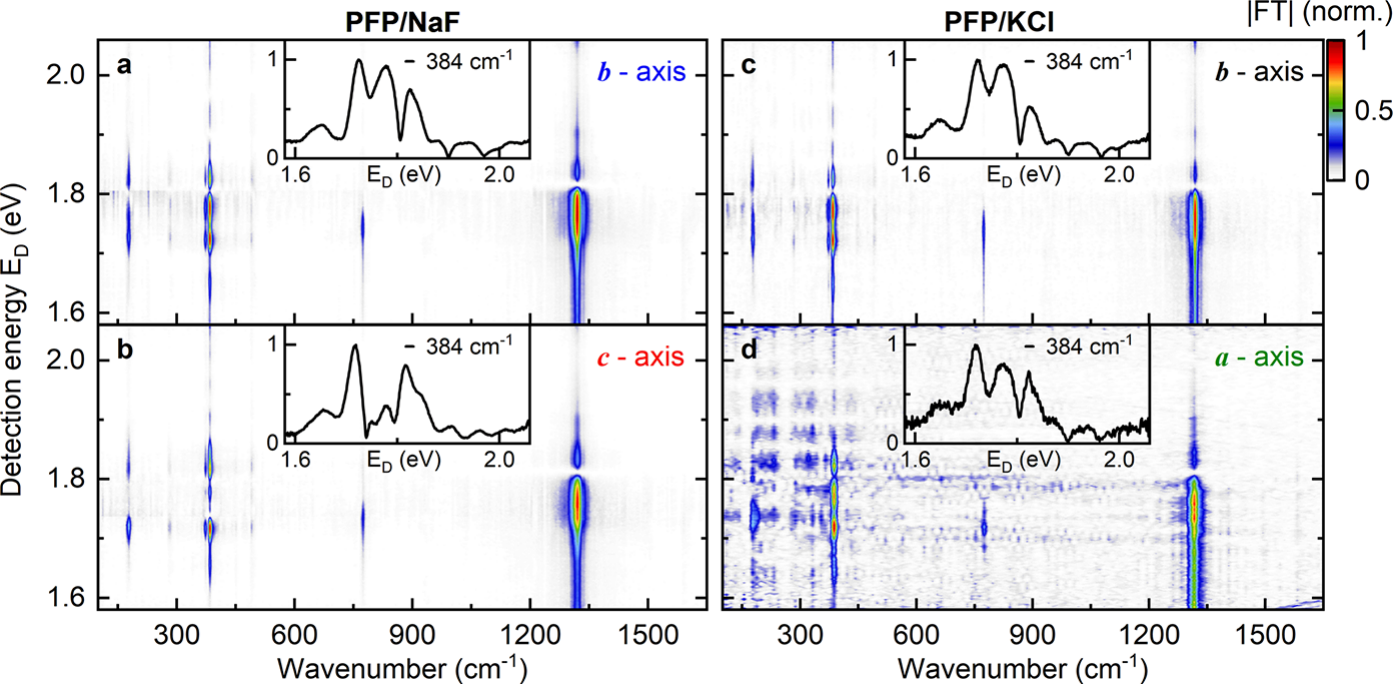
Normalized energy-resolved Fourier transform spectra obtained from the residuals of the Δ*T*/*T* maps in Figure 1e-h for excitation and probing along the (a) *b⃗*- and (b) *c⃗*-axis of PFP/NaF(100) and along the (c) *b⃗*- and (d) *a⃗*-axis of PFP/KCl(100). The amplitudes of the Fourier transform spectra are shown as a function of wavenumber and detection energy *E*_*D*_. The amplitude of the data in (d) is 10-times smaller than that in a-c since only a small portion of minority domains with *b⃗*-orientation are excited. The Fourier transform spectra are dominated by vibrational modes around 1320 cm^–1^, 774 cm^–1^, 384 cm^–1^ and 178 cm^–1^. Cross sections along *E*_*D*_ for the 384 cm^–1^ mode are displayed in the insets. Note the change in spectrum of this mode for *c⃗*-axis excitation PFP/NaF(100) in comparison to that for excitation along *b⃗*.

**Figure 5:**
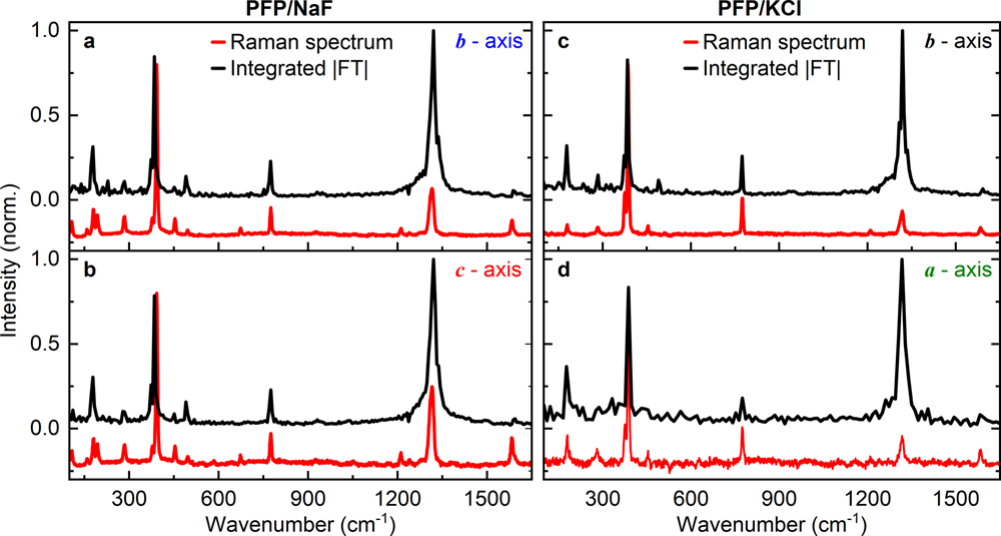
Fourier spectra (black lines), integrated along the probe energy in Figure 4 and recorded for excitation along the (a) *b⃗*- and (b) *c⃗*- axis of PFP/NaF(100) and along the (c) *b⃗*- and (d) *a⃗*- axis of PFP/KCl(100). As in Figures 1h and 2d, the amplitude of the spectrum in (d) is 10 times smaller since only a small portion of minority domains with *b⃗*-orientation are excited. Corresponding off-resonance Raman spectra recorded for linearly polarized excitation at 785 nm are shown in red. The Raman spectra have been shifted vertically by −0.3 for clarity.

We clearly identify 9 vibrational modes in the Fourier transform spectra. Their resonance frequencies and normalized amplitudes are summarized in Table 1. The resonance frequencies are reasonably close to those deduced from the Raman measurements, while the mode amplitudes that are detected in both experiments are substantially different. Since the spectral width of our excitation pulses covers the entire absorption band of PFP, the laser profile does not significantly affect the mode amplitudes.^46^ The frequencies seen in the Raman spectra agree well with those reported in ref.^32^ and with Raman measurements of PFP thin films in the π-stacked phase on graphite substrates.^33^ Most peaks, both in time-resolved and Raman spectroscopy, show line widths of less than 8 cm^–1^, corresponding to vibrational dephasing times of more than 1.5 ps. This value, partly limited by the finite scan range in the pump probe measurements and by the resolution of the Raman spectrometer, is a lower limit of the actual dephasing time. In the off-resonant Raman measurements, these narrow lines reflect long-lived vibrational wavepacket motion in the electronic ground state of the PFP crystals. We take the narrow line widths seen in the pump–probe spectra as the characteristic sign that also the persistent modulation of the pump–probe transients, lasting for up to 6 ps, probes coherent vibrational wavepacket motion in the electronic ground state of the PFP crystals.

**Table 1:** Vibrational Mode Frequencies and Amplitudes of Crystalline PFP Thin Films Deduced from Fourier Transforms of the Pump-Probe Residuals and from the Off-Resonant Raman Spectra^a^

Mode		Experimental |FT| spectrum		Experimental Raman spectrum		Experimental ref ^32^	Simulated Raman spectrum	
Mode number^30^	Symmetry^30^	Frequency (cm^–1^)	Amplitude (norm.)	Frequency (cm^–1^)	Amplitude (norm.)	Frequency (cm^–1^)	Frequency (cm^–1^)	Amplitude (norm.)
#16	Ag	178	0.32	179	0.15	178	173	0.09
#22	Ag	284	0.11	284	0.10	280	275	0.04
#36	B2g	384	0.85	392	1.00	389	396	0.01
#42	Ag	451	0.08	453	0.09	450	442	0.00
#45	Ag	490	0.14	494	0.01	492	482	0.10
#58	Ag			673	0.03	670	691	0.03
#62	B3g	774	0.23	775	0.16	770	771	0.01
#75	Ag			1210	0.03	1207	1198	0.00
#80	Ag			1238	0.00	1236	1233	0.53
#84	Ag	1320	1.00	1315	0.27	1317	1355	1.00
#86	Ag	1336	0.38			1337	1384	0.57
#99	Ag	1587	0.06	1583	0.08	1591	1573	0.10

a The experimentally measured mode frequencies are compared to values from ref (32) and from the DFT calculations performed in this work.

The coherent modulations of the pump–probe spectra are dominated by high-frequency vibrations around 1320 cm^–1^. In this frequency region, the dependence of the Fourier transform spectra on the detection energy is very similar for both substrates, and apparently does not depend on the polarization direction of the excitation laser (Figure 4). The corresponding Fourier transform spectra (Figure 5) show not only a single sharp line centered at 1320 cm^–1^ but also faint sidebands that can be interpreted as signatures of Davydov splittings.^30^ Importantly, the Fourier transform spectrum of the 1320 cm^–1^ band also displays a broadband pedestal with a width of about 30 cm^–1^. The spectral width of the pedestal reflects a faster partial decay of the 25 fs modulation on the pump–probe transients with a damping time of about 500 fs. This partial decay is also seen in Figure 3, while it is absent in the Raman spectra. We assign it to coherent vibrational wavepacket motion in an electronically excited state of the PFP crystals that is triggered by the pump laser. The line width of the off-resonant Raman spectra is defined by the dephasing time of the vibrational wavepacket motion in the electronic ground state of PFP.^47^ For off-resonant scattering, the shape of the excited state potential energy surface only affects the intensity of each Raman mode (Albrecht terms A’ and B’ in ref.^47^). In contrast, the pump laser in the transient absorption measurements is resonant with the exciton transition. It launches coherent vibrational wave packets both in the optically excited state X_S1_ and, by stimulated impulsive Raman scattering,^48,49^ in the electronic ground state. The pump–probe measurements are sensitive to the dynamics of both wave packets.^50,51^ Relaxation processes in the electronically excited state manifold may result in a rapid damping of the coherent wavepacket motion and, thus, in a broader line width in the Fourier transform spectra.^51−53^ Therefore, the pump–probe measurements allow us to distinguish excited state dynamics from vibrational ground state motion.

A similar pedestal in the Fourier transform spectra, though with reduced spectral width, is also seen for the low-frequency mode at 384 cm^–1^ (85 fs period), which causes the beating in the pump–probe residuals in Figure 3. This band, like the frequency mode, monitors not only ground state vibrations but also excited-state wavepacket motion. More importantly, for this band, the dependence of the Fourier transform spectra on the probe energy is highly sensitive to the orientation of the PFP crystals and the laser polarization. This sensitivity is illustrated in the cross sections through the Fourier transform maps along the detection energy axis at a frequency of 384 cm^–1^, shown in the insets of Figure 4 a-d. While very similar intensity profiles are seen for *b⃗*-axis excitation of PFP on both NaF and KCl, the pattern is distinctly different for *c⃗*-axis excitation of PFP on NaF(100). This is likely the most striking evidence of the effects of molecular orientation on the vibronic Davydov-splitting that we find in the present data. It is worth noting that such shifts are absent for other vibrational modes. For example, the modes at 774 and 178 cm^–1^, show the same sharp line profile in both the Fourier transform spectra and Raman spectra. For these modes, the dependence of the Fourier transform spectra on the detection energy is independent of substrate and laser polarization.

To rationalize the experimental findings, a detailed theoretical modeling of vibronic couplings in PFP crystals is necessary. This theoretical characterization should extend the DHO model, typically used to describe vibronic couplings in molecular systems. In particular, Herzberg–Teller couplings^14,54,55^ should be incorporated to account for coordinate-dependent transition dipole moments. While the theoretical framework is in principle well developed,^56−58^ its effective application requires more detailed information about the excited state potential energy surfaces of the PFP crystals. Such information can be obtained from multidimensional electronic spectroscopy^37,38,44,59^ and experiments in this direction are currently underway.

As an initial step toward interpreting the reported experiments, we present calculations of the Raman spectra of isolated PFP molecules *in vacuo* to identify the vibrational modes that mostly contribute to the measured signals. Previous calculations of the infrared-active modes of PFP crystals^30^ indicated a resemblance with the vibrational modes obtained for an isolated PFP molecule in vacuo, except for expected Davydov splitting of some of the modes and small energetic shifts. This analogy confirms that single molecule calculations can provide an accurate mode assignment.

The vibrational modes of an isolated PFP molecule in vacuum are calculated in the harmonic approximation in the framework of density-functional theory.^60^ For computational details, see the Methods section. The IR spectrum reported in Figure 6a (gray shaded area) indicates a predominant activity in the high-frequency region above 800 cm^–1^ where the C–C modes dominate. This result agrees well with the one reported in ref.^30^ The calculated Raman spectrum of the PFP molecule (Figure 6b, red curve) is also dominated by an intense activity in the high-frequency region (1200–1600 cm^–1^) where the deformation modes of the benzene rings have their normal frequencies. These modes are ubiquitous in carbon-conjugated molecules and appear with similar intensity also in pentacene and other oligoacenes.^61^

**Figure 6:**
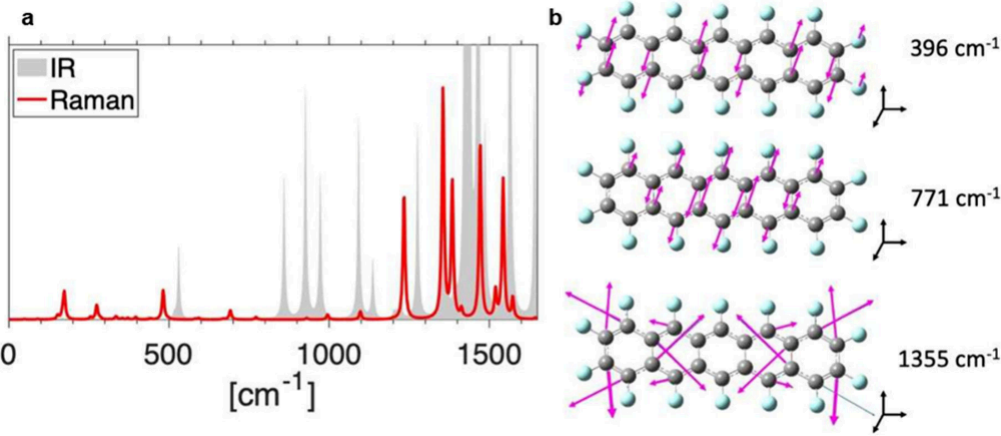
(a) Infrared (IR, gray area) and resonant Raman spectra (red) of an isolated PFP molecule in vacuo calculated using DFT with the PBE functional. (b) Visualization of selected Raman active modes. C atoms are shown in gray and F atoms in cyan. The arrows indicate the directions of the atomic displacements. Both spectra are broadened by a Lorentzian function with a fwhm of 5 cm^–1^.

The calculated Raman spectrum, however, partially contrasts with the experimental data (Figure 5), where only a single strong peak at ∼1320 cm^–1^ and a faint sideband at 1336 cm^–1^ are seen. The other Raman-active peaks predicted by the single-molecule calculation are absent in the measurements. This behavior can be understood considering the stiffness of the crystalline arrangements in which the molecular backbones hosting the C–C modes have considerably fewer degrees of freedom compared to the gas-phase. The results of the calculations indicate that the 1320 cm^–1^ mode is a symmetric, in-plane deformation of the fused carbon rings in which the C atoms predominantly move along the short axis of the molecule, corresponding to the direction of the HOMO–LUMO transition dipole moment of the PFP molecule (Figure 6b). The lowest energy excitation in the crystalline phase shares the same intramolecular polarization, explaining why the 1320 cm^–1^ mode governs the vibronic coupling of the *X*_*S*__1_ excitons in the single crystals. Nearest-neighbor coupling between electronic transition dipole moments not only delocalizes the electronic excitation, forming two delocalized Davydov-split excitonic states with transition dipole moments oriented along the *b⃗*- and *c⃗*-axes; it also results in the formation of delocalized symmetric (*Q*_+_) and antisymmetric (*Q*_–_) vibrational modes by in-phase and out-of-phase superposition of the 1320 cm^–1^ mode of neighboring PFP molecules in the unit cell.^17,40,56^ The variation of the potential energy of the two excitons along the *Q*_+_ and *Q*_–_ modes may be most relevant for the wavepacket dynamics observed in the pump–probe experiments. Such an interpretation of the absorption spectrum of the PFP crystals may explain the detection energy dependence of the Fourier transform spectra reported in Figure 4.

The low-frequency region of the vibrational spectrum of PFP (150–500 cm^–1^) is characterized by C–F modes delocalized across the molecule and involving the motion of the heavier fluorine atoms. The C–F modes exhibit a lower relative intensity compared than the C–C ring deformation modes at higher frequencies (see Figure 6a). An exception is the mode at 396 cm^–1^, seen with low intensity in the simulations but with high intensity in the experimental spectra at 384 cm^–1^. We reiterate that such energetic discrepancies are expected and do not invalidate this comparison. This mode is connected to an out-of-plane ring bending visualized in Figure 6b. The comparison to the experimental data suggests that it is this out-of-plane bending motion that results in a vibronic coupling that is sensitive to the molecular orientation and, thus, induces the polarization-dependent beating pattern for the PFP film on NaF(100) that is seen in Figure 3b,c and Figure 4a,b.

The experimental spectra suggest that two additional modes at 178 and 774 cm^–1^ play a role in the dynamics. For both modes, the vibronic couplings observed in the experiment do not depend on the crystal orientation. The calculations reveal a Raman-active mode at 173 cm^–1^, which is a symmetric low-frequency stretching mode of the molecule involving also the motion of the fluorine atoms. On the other hand, the calculated Raman-active mode that appears at 771 cm^–1^ is an out-of-plane bending mode (Figure 6b).

Overall, the comparison between experiments and simulations suggests that the single-molecule calculation is not able to fully describe the vibronic complexity detected in the crystalline samples. Only the inclusion of long-range Coulomb interactions and of the periodic character of the wave functions in the model can lead to a quantitative agreement with the measurements and hence, to a convincing interpretation of the experimental observations. For the same reason, a simplified model including only a PFP dimer is likely equally unable to rationalize the vibronic couplings between the involved electronic transitions as well as to explain wave packet dynamics and detection energy dependence of the Fourier transform spectra. To achieve these goals, full-fledged simulations of the crystalline phase are required.^61,62^ Despite these limitations, the presented calculations on the isolated PFP molecules suggest that in-plane and out-of-plane deformation vibrations of the carbon backbone play a dominant role for the vibronic couplings in the crystals and provide important qualitative indications to interpret the experiments in this direction.

In summary, we investigated coherent vibronic couplings to excitons of crystalline perfluoropentacene thin films epitaxially grown with different molecular orientation (lying vs standing) on KCl(100) and NaF(100) substrates. Our experimental results, combining ultrafast pump–probe spectroscopy with 10 fs time resolution and Raman spectroscopy, demonstrate different effects of vibronic couplings on the optical properties of these thin films. The pump–probe spectra show a Davydov splitting of ∼30 meV between the lowest-lying exciton transitions with dipole moments oriented perpendicular to the long axis of the PFP molecules. Persistent high-frequency oscillations of the pump–probe transients with a 25 fs period point to a predominant coupling of the excitonic transitions to a symmetric ring deformation mode of the carbon backbone with a dimensionless displacement of ∼0.6 that is insensitive to the molecular orientation on the film. Couplings to higher-frequency modes are largely suppressed. Vibronic couplings to a lower-frequency out-of-plane ring bending modes give rise to a characteristic interference pattern in the pump–probe transients that changes when varying the relative orientation between laser polarization and crystal axes. The polarization-dependence provides evidence that the coupling between excitons and this out-of-plane mode depends sensitively on the molecular orientation in the thin film. This points to a significant role of the molecular arrangement for the vibronic couplings and, therefore, potentially also for the fission of singlet excitons in these samples. DFT calculations of the Raman spectra of isolated PFP molecules *in vacuo* aid in assigning the relevant vibrational modes, although additional analysis including long-range Coulomb interactions and a detailed modeling of the crystalline samples, planned for upcoming work, is necessary for a complete interpretation of the measurements.

In essence, our results show that single-crystalline thin films of perfluoropentacene, or acenes in general, appear as interesting model systems for exploring the potential role of vibronic couplings and their dependence on molecular orientation on the dynamics and yield of singlet fission processes. They suggest that polarization-controlled time-resolved pump–probe and multidimensional coherent spectroscopies with 10 fs time resolution can sensitively probe coherent couplings to even the fastest vibrational modes in the organic crystals and their dependence on the molecular orientation. The present study demonstrates the ability of ultrafast time-resolved spectroscopy to detect coherent couplings of vibrational modes and electronic excitations in crystalline organic thin films and paves the way to analyze the role of vibronic couplings on singlet exciton fission processes.

## Methods

### Sample Preparation and Sample Growth

The PFP (Kanto Denka Kogoyo, purity >99%) films are grown on crystalline NaF (001) and KCl (001) surfaces following the growth procedure described in ref.^10^ The alkali halide surfaces are prepared by cleaving slices of about 2 mm thickness from a single-crystal rod (Korth Kristalle GmbH) in air. After transfer into the vacuum system, the substrates are annealed at 450 K to remove adsorbed water. Subsequently, the highly crystalline PFP thin films (100−200 nm) are prepared under ultrahigh-vacuum conditions by molecular beam deposition at a molecular flux of about 6 Å/min as monitored by a quartz crystal microbalance. To maximize the domain sizes, the PFP films are grown at a substrate temperature of 350 K. PFP forms epitaxially ordered adlayers of the bulk structure. On NaF(100) substrates, the molecules adopt an upright orientation with their *b⃗*- and *c⃗*-axes parallel to the surface. On KCl, the PFP molecules adopt a recumbent orientation, yielding the *a⃗*- and *b⃗*-axes parallel to the substrate surface. The crystalline structure of all samples is verified by X-ray diffraction, optical microscopy, and atomic force microscopy as detailed in ref.^10^

### Coherent Vibrational Spectroscopy

For ultrafast and broadband coherent vibrational spectroscopy^63,64^ (CVS) of PFP samples we use a home-built noncollinear optical parametric amplifier (NOPA)^36^ that is based on the design in ref.^65^ The NOPA is pumped by a Carbide (Light Conversion) laser system operated at 200 kHz repetition rate. The output spectrum of the NOPA is shown in Figure S3b, as recorded using a fast and sensitive line camera (Octoplus, e2v) with an acquisition rate of 100 kHz. The normalized root-mean square error (NRMSE) of 10000 consecutively recorded spectra at an acquisition rate of 100 kHz shows a high spectral stability of the NOPA that is mostly limited by detector shot-noise (see Figure S3b).

These NOPA pulses are compressed using chirped mirrors (DCM9, Laser Quantum) and used in a pump–probe setup.^36,38^ Using a beam splitter, the same laser spectrum is used as the pump and the probe. An in-line interferometer based on birefringent wedges (TWINS),^66^ used to generate a phase-stable excitation pulse pair, is set to a delay of zero. Additional dispersion caused by the TWINS is compensated for by using a second pair of chirped mirrors (DCM12, Laser Quantum) in the pump arm of the setup. The time delay *t*_*d*_ between pump and probe is tuned via a motorized linear translation stage (M126.DG, Physik Instrumente). Both pump and probe are focused onto the sample using an off-axis parabolic mirror (OAP) to a spot size of around 30 × 30 μm^2^ at their intersection. Both beams are polarized along the same direction. A broadband achromatic half-wave plate (B. Halle) is employed to simultaneously tune the polarization of the pump and probe directly before the OAP such that both beams are always polarized in the same direction. Using a 10-μm-thick beta barium borate crystal, a cross-correlation second-harmonic frequency resolved optical gating (SH-FROG) is measured between the pump and the probe at the sample position. The measured FROG trace in Figure S3a yields a retrieved pulse duration of ∼10 fs.

In the experiments, the PFP samples are placed behind the OAP and investigated in transmission geometry. The transmitted probe is sent to a grating spectrograph (Acton SP2150i, Princeton Instruments) with an attached line-camera (Octoplus, e2v) operating at 100 kHz, half of the laser repetition rate. Fast mechanical chopping of the pump is achieved by using an optical chopper system (MC2000B, Thorlabs) that uses a custom-made wheel with 500 slots. The pump is therefore chopped at 50 kHz such that the line camera can record spectra with (*S*_*on*_) and without (*S*_*off*_) presence of the pump, each in pairs of two laser pulses. From each pair of two consecutive spectra we compute the differential transmission
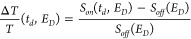
1as a function of the pump–probe delay *t*_*d*_ and the probe energy *E*_*D*_. For each delay, 5000 consecutive differential spectra are recorded and averaged. A step-size of 3 fs is used, and delays are scanned up to 6 ps for PFP on NaF. For PFP on KCl, the time delays are scanned up to 6 ps when the laser pulses are polarized along the *b⃗*-axis and up to 3 ps along the *a⃗*-axis of crystal. To increase the signal-to-noise ratio, multiple of such scans are repeated and averaged afterward.

All experiments are performed at room temperature and under ambient conditions. The half-wave plate is used to tune both pump and probe to align with the desired crystal orientation. Experiments are performed with a pump fluence of 800 μJ/cm^2^ for the sample on NaF substrate and 730 μJ/cm^2^ for KCl substrate. The probe is set to 330 μJ/cm^2^. During experiments, no signs of sample degradation have been observed. A study of the effect of the pump fluence on the pump–probe signal (Figure S4) ensured that all experiments are performed within the regime of χ^(3)^ nonlinearities.

### Raman Spectroscopy

Raman spectra of PFP/NaF(100) and PFP/KCl(100) were recorded using a WITec Raman microscope equipped with a UHTS-300 SMFC visible spectrometer with a 300 g/mm grating blazed at 750 nm. The excitation wavelength was 785 nm, and the incident laser was linearly polarized with a power set to 50 mW. The excitation beam was focused on the sample to a spot size of approximately one micron with a microscope objective with numerical aperture (NA) of 0.4 and 20× magnification. To record polarization-dependent Raman spectra, the polarization was varied from 0° to 350° in increments of 10° using a half-wave plate.

### Analysis of Pump–Probe Data

To analyze the oscillatory modulation in the differential transmission Δ*T*/*T* maps, we isolated the oscillatory part by subtracting the slow varying background dynamics characterized by multiple exponential decays. The resulting residual maps, shown in Figure 2 of the manuscript, were normalized to the maximum Δ*T*/*T* signal at delay time of 120 fs. This process was applied to all data sets. Figure S5 illustrates the procedure for PFP/NaF, where the laser is polarized along the *b⃗*-axis.

Subsequently, we calculated the Fourier transform (FT) of the residuals at each detection energy to obtain the energy-resolved Fourier transform spectra for PFP on NaF and KCl that are shown in Figure 4. The resolution of the FT map, determined by the measurement time, is 6 cm^–1^ for PFP/NaF along both the *b⃗*- and *c⃗*-axis, as well as for the *b⃗*-axis of PFP/KCl. For the *a⃗*-axis of PFP/KCl, the resolution is 12 cm^–1^.

### Calculation of IR and Raman Spectra

The *ab initio* simulations of Raman spectra on PFP molecules *in vacuo* were carried out in the framework of DFT as implemented in the software Gaussian 16^67^ using the PBE functional^60^ and cc-pVTZ basis set. Geometry optimization of the isolated PFP molecule was performed without symmetry constraints and with a threshold for the interatomic forces of 10^–8^ Hartree/bohr. Vibrational modes were calculated in the harmonic approximation. The intensity of the IR-active modes depends on the derivative of the dipole moment μ with respect to the normal coordinates *Q*_*n*_, according to *I* ∝ , while the Raman cross-section along a specific scattered direction follows the derivative of the electronic polarizability tensor *χ*_*ij*_ with respect to the normal coordinates *Q*_*n*_, *I* ∝ , with *e*_*I*__,*i*_ (*e*_*S*__,*i*_) being the *i*-th polarization of the incident (scattered) electric field. For more details about this theory, we redirect readers to refs.^68,69^ Discrepancies of the order of a few cm^–1^ between calculated and measured frequencies are expected and do not hamper the comparison between experiment and theory.

## Data Availability

All data supporting the findings are presented in the Letter and Supporting Information in graphic form. Experimental raw data and simulation results will be provided by the authors upon reasonable request.

## Supporting Information Available

X-ray diffraction of PFP films on KCl and NaF; polarization-resolved UV/vis absorption spectra of single crystalline PFP islands on KCl and NaF; pulse characterization; fluence study; data analysis (PDF)
Transparent Peer Review report available (PDF)
