# Supplementary material for: Ultrafast Time-Domain Spectroscopy Reveals Coherent Vibronic Couplings upon Electronic Excitation in Crystalline Organic Thin Films
Source: J Phys Chem Lett. 2024 Oct 31;15(44):11170–81. doi: 10.1021/acs.jpclett.4c02711 (PMC11552073; doi:10.1021/acs.jpclett.4c02711)
Supplement: Supplementary file 1 — jz4c02711_si_001.pdf [file jz4c02711_si_001.pdf]

Supporting Information for

Ultrafast Time-Domain Spectroscopy Reveals Coherent Vibronic Couplings Upon Electronic Excitation in Crystalline Organic Thin Films

Somayeh Souri[a], Daniel Timmer[a], Daniel C. Lünemann[a], Naby Hadilou[a], Katrin Winte[a], Antonietta De Sio[a,c], Martin Esmann[a,c], Franziska Curdt[b], Michael Winklhofer[b], Sebastian Anhäuser[d], Michele Guerrini[a], Ana M. Valencia[a], Caterina Cocchi[a,c], Gregor Witte[d], Christoph Lienau[a,c,e*]

[a] Institut für Physik, Carl von Ossietzky Universität, Carl-von-Ossietzky Str. 9-11, 26129 Oldenburg, Germany

[b] Institut für Biologie, Carl von Ossietzky Universität, Carl-von-Ossietzky Str. 9-11, 26129 Oldenburg, Germany

[c] Center for Nanoscale Dynamics (CENAD), Carl von Ossietzky Universität, Carl-von-Ossietzky Str. 9-11, 26129 Oldenburg, Germany

[d] Fachbereich Physik, Philipps-Universität Marburg, Renthof 7, 35032 Marburg, Germany

[e] Research Centre for Neurosensory Sciences, Carl von Ossietzky Universität, Carl-von-Ossietzky Str. 9-11, 26129 Oldenburg, Germany

*Correspondence to: christoph.lienau@uni-oldenburg.de

1. X-ray diffraction of PFP films on KCl and NaF

In order to determine the out-of-plane orientation of PFP films on KCl and NaF, X-ray diffractograms were measured in Bragg-Brentano-geometry, using a Bruker D8 Discovery diffractometer equipped with a Göbel mirror yielding monochromatized Cu Kα radiation (λ =

1.542 Å) and a sensitive one-dimensional LynxEye silicon strip detector. The obtained diffractograms are shown in Figure S1. In the case of PFP on KCl (Figure S1a), an intense reflection corresponding to the (102) plane in the PFP bulk crystal structure can be observed, which is in line with the results from previous work.[1] The schematic images in Figure S1b show the molecular orientation relative to this plane from a top and a side perspective. The PFP molecules are recumbently oriented to the KCl substrate, with an inclination of about 8° of the molecular L-axis to the surface. The diffractogram also features a very tall peak at higher diffraction angles, originating from the single-crystalline KCl substrate. Because the signal from the substrate is so intense, the curve was scaled down by a factor of 5000 here. Another consequence of the strong diffraction from the substrate is the occurrence of artefact peaks due to the nonperfectly monochromatized X-ray spectrum. Parasitic wavelengths are also scattered by the (200) plane of the KCl substrate and contribute additional peaks. On the one hand, the Cu Kβ line is not entirely cancelled out by the nickel-filter, which gives rise to a sharp peak next to the Kα signal of the KCl (200) plane. On the other hand, the Göbel mirror transmits not only the Cu Kα line, but also 1/3 of this wavelength, resulting in the λ/3-peak as indicated in the figure. The diffractogram also features a small peak at $2\Theta=5.7°$, corresponding to the (100) plane of the PFP crystal structure. Since this peak is relatively small compared to the dominating (102) peak, even though the structure factor of the (100) reflex is four times higher, we attribute this to defect-induced growth of PFP at imperfect sites on the KCl crystal such as steps.

In the case of PFP on NaF, the (100) peak and its higher-order equivalents are very pronounced in the diffractogram in Figure S1c. As shown in the schematic images in Figure S2d, the (100) plane corresponds to an upright standing molecular orientation of the PFP film on the substrate with a small angle of 14° between the molecular L-axis and the surface normal. Again, the single-crystalline substrate gives rise to an intense reflex as well as the parasitic λ/3- and Kβ-peaks.

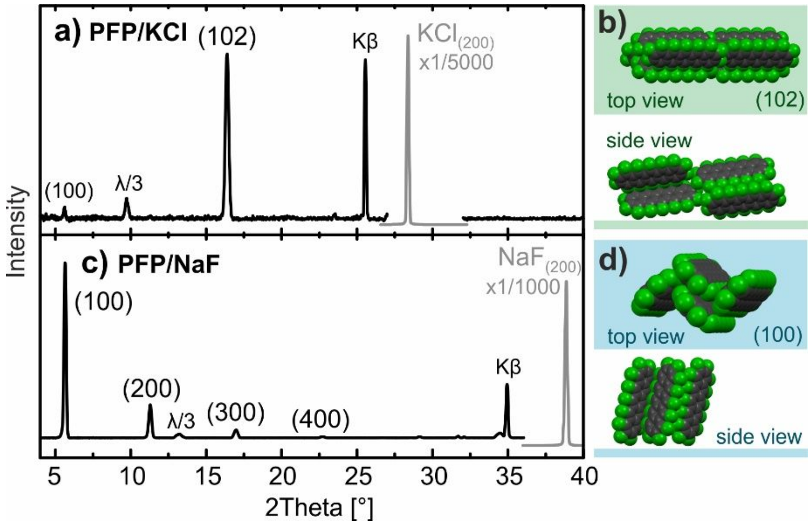

Figure S1. X-ray diffractograms of PFP films on a) KCl and c) NaF. On KCl, PFP films are (102)-oriented, corresponding to a recumbent molecular orientation shown schematically in b). On NaF, the PFP molecules adopt an upright standing orientation with the (100) plane parallel to the substrate as seen in d).

2. Polarization-resolved UV/Vis absorption spectra of single crystalline PFP islands on KCl and NaF

PFP films on KCl and NaF exhibit strong differences in their optical properties, which can be seen already from their overall color appearance. While PFP films on KCl have a green color,

films on NaF appear blue when viewed from the top. To quantify this effect, polarization-resolved UV/Vis spectra of individual single crystalline islands were taken in transmission geometry using an optical polarization microscope with an attached UV/vis spectrometer yielding a spot size of about 20 μm and exploiting the optical transparency of the alkali halide substrates. The obtained spectra as well as polarized optical micrographs are shown in Figure S2. The spectra of PFP on KCl in Figure S2a feature two main absorption bands: One lower-energy absorption band around 1.75eV and one higher-energy absorption band at 2.75eV, each accompanied by vibronic replicas. From a multi-Gaussian fit (dashed lines), we determined the energetic difference between the lowest-energy "M"-excitation and its first vibronic replica to be hν = 166 meV (1339 cm^{-1}) which is in close agreement with the vibrational mode energy derived from time resolved pump-probe spectroscopy and from Raman spectroscopy. When the polarization of the incident light is rotated around the azimuth, the intensities of the two absorption bands change periodically, as depicted in the inset in Figure S2a. More precisely, the peak intensities follow a $\sin^2$ function with a 90° phase shift with respect to each other. This can be explained when taking the molecular orientation and the transition dipole moments (TDMs) of the optical excitations into account. From section S1 we know that the PFP molecules are recumbently oriented on the KCl substrate (cf. Figure S1b). On the other hand, DFT calculations have shown that the TDMs of the molecular optical excitations, which give rise to the excitonic resonances seen in the spectra, are polarized along the molecular M-axis in the case of the lower-energy bands and along the L-axis in the case of the higher-energy bands.[2] Since the intensity of absorption is proportional to $\left|\vec{E} \cdot \overrightarrow{TDM}\right|^2$, the overlap of the electric

field vector $\vec{E}$ with the respective molecular axis is responsible for the absorption intensity. In the given recumbent molecular orientation, both the M- and L-polarized bands can be excited and, because of the collinear alignment of the L-axes in the crystal structure, the L-polarized bands are maximal when the M-polarized ones have their minimum, and *vice versa*.

Looking closely, the intensities of those bands never reach zero. This implies that even within a domain where the majority of PFP molecules share the same azimuthal orientation, a contribution from small minority domains of PFP molecules, rotation in orientation by 90°, remains. Therefore, a residual absorption of the M-polarized band is seen even if the light is polarized along L, and the other way around. In the projection onto the (012) plane, the polarization along M and L is equivalent to the unit cell vectors $\vec{b}$ and $\vec{a}$, respectively, as depicted in Figure S2a. The strong absorption anisotropy can also be seen nicely in the polarized optical micrograph in the right panel, where single-crystalline domains with different azimuthal orientations show either a dark blue or a yellow color, depending on the relative orientation between $\vec{E}$ and $\vec{b}$ ($\vec{a}$). Hence, also the aforementioned minority domains inside larger majority domains of one preferential azimuthal orientation can actually be visually seen because of their different color in the micrograph inside the exemplary measurement spot, drawn as a white circle.

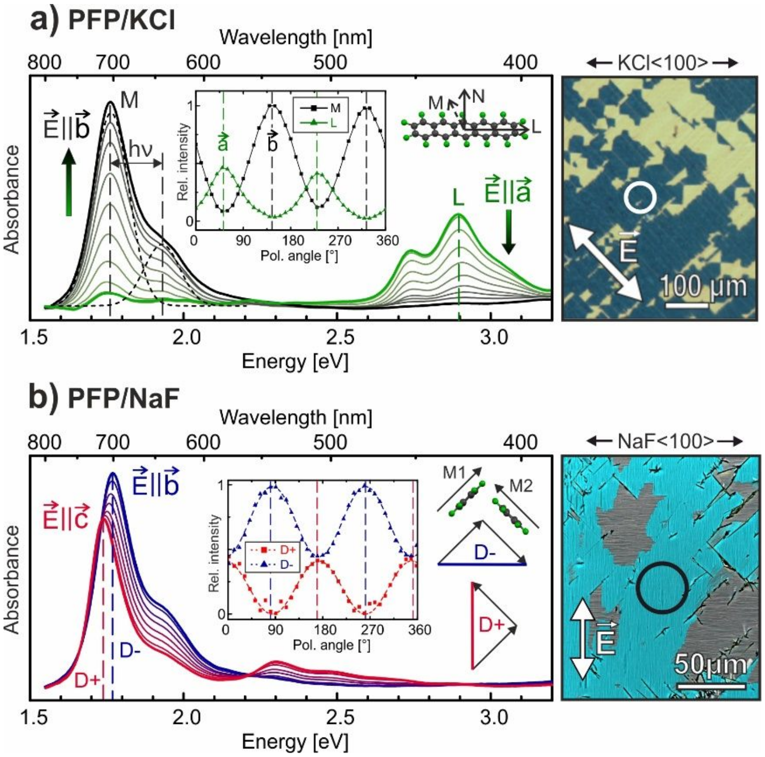

Figure S2. Polarization-resolved UV/Vis absorption spectra of PFP films on a) KCl and b) NaF. The different molecular orientations give rise to optical anisotropies of the M- and L-polarized bands in (a). A vibronic side peak of the M band is shifted in energy from the main transition by $h\nu$, as depicted by dashed lines. For PFP on NaF (b), a Davydov-splitting between the bands polarized along $\vec{b}$ (D^{-}) and $\vec{c}$ (D^{+}) is marked by dashed lines (b). Polarized optical micrographs (right panels) show the different color appearances of single-crystalline domains within the films. Optical spectra have been recorded at the spots marked by open circles.

In the case of PFP on NaF, the spectra look different, as shown in Figure S2b. Here, the L-polarized bands are not visible at any polarization angle. This can be explained by the fact that in the upright standing (100)-orientation, the overlap of the L-polarized TDM with $\vec{E}$ is insignificant. However, the lower-energy absorption band exhibits a fine structure which could not be seen for PFP on KCl. This can be attributed to Davydov-splitting of about 30 meV, where the M-polarized transition dipole moments M1 and M2 of two inequivalent molecules within the unit cell can form linear combinations, giving rise to a D^{+} and a D^{-}-component, as illustrated in the inset of Figure S2b. The resulting excitations are polarized along $\vec{c}$ and $\vec{b}$, respectively. Therefore, they show a

phase difference of 90° in the polarization-resolved measurement, which is also shown in the inset. Since the two Davydov-components spectrally overlap, multi-Gaussian fits were employed for each spectrum and the respective amplitudes of the Gauss fits are plotted against the polarization angle. This 90° phase contrast of the two Davydov-components gives rise to the blue and reddish color impressions of single-crystalline PFP domains with different azimuthal orientations, which are shown in the polarized optical micrograph in Figure S2b in the right panel. The same Davydov-splitting also occurs in the PFP films on KCl, since they share the same crystal structure. However, for geometrical reasons, only the D^{-} component can be seen there.

3. Pulse characterization

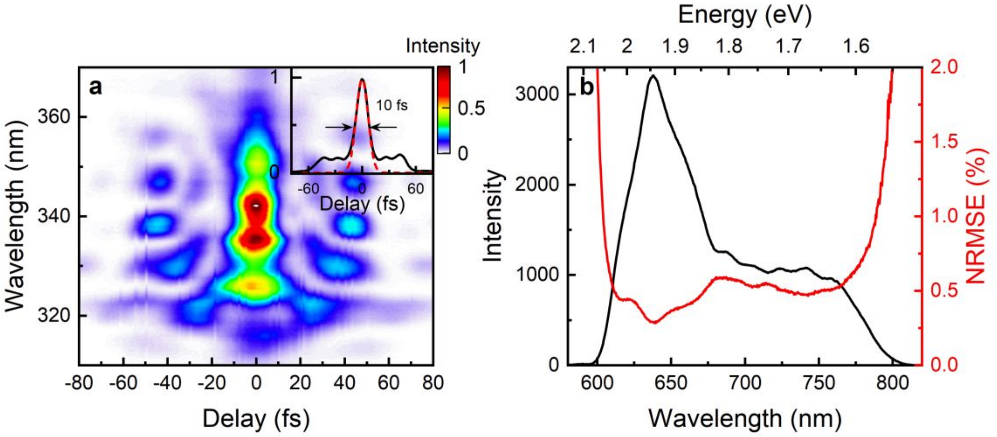

Figure S3. (a) Second harmonic frequency resolved optical gating (SH-FROG) measurement of the cross correlation between pump and probe pulses at the sample position. The inset shows the retrieved temporal intensity of the laser pulses with a full-width at half-maximum (FWHM) duration of 10 fs. The retrieved pulse duration is ~10 fs. (b) NOPA spectrum and stability. The normalized root-mean square error (NRMSE) is computed from 10000 consecutively recorded spectra acquired with a rate of 100 kHz.

4. Fluence study

To ensure that all experiments are performed within the linear regime of $\chi^{(3)}$ nonlinearities[3], we study the dependence of the pump-probe signal of PFP on NaF on the pump fluence for delays up to 1 ps. For this, the pump fluence is tuned from 100 μJ/cm² to 800 μJ/cm², while the probe is fixed at 330 μJ/cm². The resulting normalized dynamics at the maximum position of the resonance and differential spectra at a delay of 300 fs are presented in Figure S4a,b and do not show any effect on the pump fluence. The extracted signal amplitude, taken as the average signal at $E_D = 1.779$ eV for delays between 500 fs and 1000 fs, is shown in Figure S4c. For the investigated range of pump fluences, no deviation from a linear behavior can be observed.

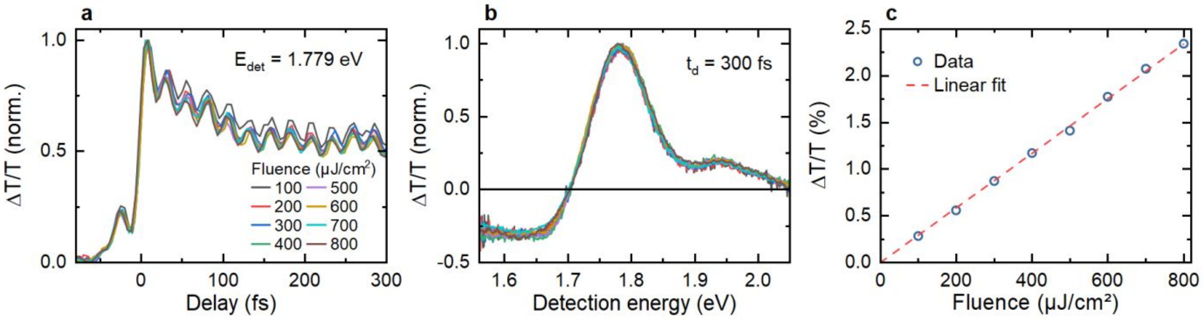

Figure S4. Fluence study of PFP on NaF for pump fluences ranging from 100 μJ/cm² up to 800 μJ/cm² using a probe fluence of 330 μJ/cm². (a) Normalized dynamics of the first 300 fs for the different fluences taken at a probe energy of $E_D = 1.779$ eV, the maximum position of the main resonance in the $\Delta T/T$ spectra. (b) Normalized differential transmission spectra taken at a delay of $t_d = 300$ fs. **c:** Amplitude of the differential transmission signal at the maximum position ($E_D = 1.779$ eV), averaged from 500-1000 fs delay (blue circles), together with a linear fit (red line). No deviation from a linear behavior within the experimental fluence range can be observed.

5. Data analysis

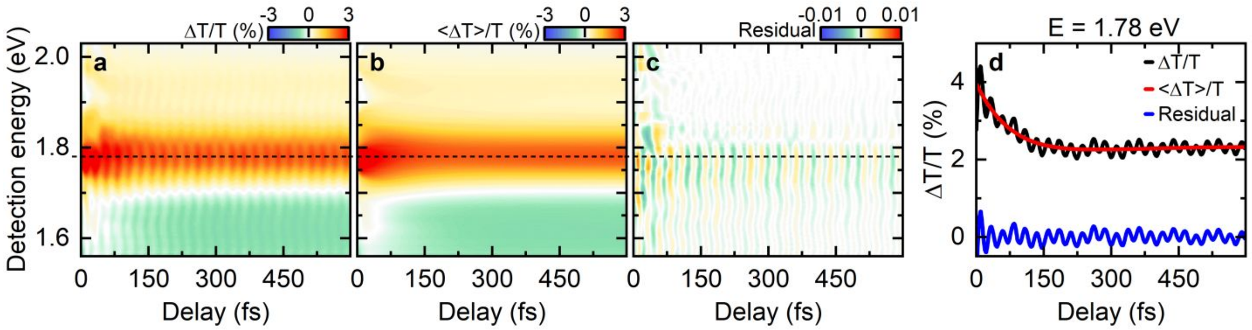

Figure S5. (a) Differential transmission $\Delta T/T$ map recorded for laser polarization along the b-axis of PFP/NaF. (b) Corresponding slow-varying temporal dynamics of differential transmission map, $<\Delta T>/T$. (c) Residual maps obtained by subtracting b from a, highlighting the oscillatory modulation in the $\Delta T/T$ map. (d) Cross-sections of the $\Delta T/T$, $<\Delta T>/T$ and residuals at a detection energy of 1.78 eV, indicated by the dashed lines in (a–c).

6. References

(1) Breuer, T.; Witte, G. Epitaxial growth of perfluoropentacene films with predefined molecular orientation: A route for single-crystal optical studies. Physical Review B 2011, 83 (15).

(2) Valencia, A. M.; Bischof, D.; Anhäuser, S.; Zeplichal, M.; Terfort, A.; Witte, G.; Cocchi, C. Excitons in organic materials: revisiting old concepts with new insights. Electronic Structure 2023, 5 (3), 033003.

(3) Mukamel, S. Principles of nonlinear optical spectroscopy; Oxford University Press, 1995.
