# Supplementary material for: Ultrafast Time-Domain Spectroscopy Reveals Coherent Vibronic Couplings upon Electronic Excitation in Crystalline Organic Thin Films
Source: J Phys Chem Lett. 2024 Oct 31;15(44):11170–81. doi: 10.1021/acs.jpclett.4c02711 (PMC11552073; doi:10.1021/acs.jpclett.4c02711)
Supplement: Supplementary file 2 — jz4c02711_si_002.pdf [file jz4c02711_si_002.pdf]

jz-2024-02711y.R1

Name: Peer Review Information for "Ultrafast Time-Domain Spectroscopy Reveals Coherent Vibronic Couplings Upon Electronic Excitation in Crystalline Organic Thin Films"

First Round of Reviewer Comments

Reviewer: 1

Comments to the Author

Dear Editor,

I have reviewed the paper on coherent oscillations in perfluoro pentacene by Souri et al. In brief, I think it is a good quality ultrafast study of impulsive Raman modes and is presented in a detailed scholarly way. All of the necessary background and control experiments (e.g. eliminating the role of the substrate) have been performed. It may seem that it is yet another vibrational wavepacket analysis of a solid organic molecule, but note that in contrast to most previous studies of the same system, these have been done on single crystals with well-known crystalline geometries (induced by the substrate) and crystal domains that are larger than the focus spot size. The contrast between the two different crystal orientations clearly show that the origin of the oscillations is intermolecular, thereby giving unique insight that is otherwise ambiguous from other experiments.

I recommend its publication and believe that it will serve the organic electronics community, especially in the context of singlet fission, as a good benchmark for assessing impulsive vibrations.

Reviewer: 2

Comments to the Author

Souri et al present an ultrafast spectroscopy study of perfluoropentacene, focusing on the vibrational coherences generated through impulsive excitation. This is not a new technique, but advances in instrumentation have given it new currency and it is gaining renewed attention, including in the analysis of singlet fission (relevant to PFP here). One of the key aspects missing so far in the analysis of vibrational coherences is the correlation between the vibronic couplings that they report on and molecular packing. The authors use a creative approach here of growing large crystalline domains with the same packing but different orientations, on two different substrates, permitting VC analysis with polarization control that probes different coupling directions. The

experimental work is of high quality, and the analysis is very well described. The study demonstrates the analytical power of this VC technique, especially when applied to crystalline materials, to probe the dynamics of vibronic coupling. It should be a significant interest to the ultrafast spectroscopy community and establishes a good model to follow, and I recommend publication. I raise a few minor issues below that would improve the manuscript.

1. The authors make reference to the UV-Vis spectra reported in reference 30. I would strongly encourage the authors to provide their own (ideally polarization-resolved) UV-Vis spectra; such a basic characterization is quite important in interpreting time-resolved spectroscopy results. This is even more the case when factoring in the pulse overlap parameters that impact vibrational coherence spectra. It is needlessly cumbersome for the reader to have to resort to a different publication to find the UV-Vis spectrum.
2. Page 25, line 35: '…suggest this is this out-of-plane…' is presumably missing an 'it'
3. Based on the literature cited, there are different practices in the field for handling such vibrational coherence data; it would be useful for the authors to provide additional details about the data processing such as the use (or not) of window functions.
4. There has been significant theoretical work on the interpretation of such vibrational coherences from Andrzejak and Petelenz (DOI: 10.1021/acs.jpcc.9b11772, DOI: 10.1021/acs.jpcc.0c06504 & DOI: 10.1021/acs.jpcc.4c01485). Among other things, these papers indicate that quantitative analysis of the vibrational coherences requires explicit consideration of the excitation pulse profile and its overlap with the absorption. Has this been factored into the FFT intensity analysis, or should it not matter given the pulse overlap used in these experiments?

Author's Response to Peer Review Comments:

Prof. Dr. Christoph Lienau
Institut für Physik
Carl von Ossietzky Universität
26129 Oldenburg Germany

Oldenburg, 18.10.2024

Senior Editor
The Journal of Physical Chemistry Letters

Revisions for Manuscript ID: jz-2024-02711ynl-2024-032406 for JPCL

Dear Editor,

We are glad to hear the positive reviews for our manuscript, supporting publication of our manuscript in the Journal of Physical Chemistry Letters after minor revision. In our revised manuscript we tried to address

all questions and comments of the Reviewers and included all necessary changes in the manuscript. Our respective changes are summarized below. In response to a request of one of the Reviewers, we have recorded new X-ray diffraction data and polarization-resolved UV/Vis spectra of our samples. These data are now put in two new sections in the Supporting Information. We suggest to add the Ph.D. student Sebastian Anhäuser as a co-author of our manuscript.

We hope that the paper now meets all criteria for publication in JPCL. We also want to thank you for the editorial handling of our manuscript.

With my best regards,

Christoph Lienau

Point-by-point response to the Reviewers.

We appreciate the Reviewers' thoughtful evaluation of our work and their constructive comments and suggestions, helping us to improve the manuscript. In the following, we provide detailed, point-by-point responses to their concerns. We also highlight the specific changes made to the revised manuscript and supplementary information in red.

Reviewer #1 (Comments for the Author):

This paper represents a significant new contribution and should be published as is.

I have reviewed the paper on coherent oscillations in perfluoro pentacene by Souri et al. In brief, I think it is a good quality ultrafast study of impulsive Raman modes and is presented in a detailed scholarly way. All of the necessary background and control experiments (e.g. eliminating the role of the substrate) have been performed. It may seem that it is yet another vibrational wavepacket analysis of a solid organic molecule, but note that in contrast to most previous studies of the same system, these have been done on single crystals with well-known crystalline geometries (induced by the substrate) and crystal domains that are larger than the focus spot size. The contrast between the two different crystal orientations clearly show that the origin of the oscillations is intermolecular, thereby giving unique insight that is otherwise ambiguous from other experiments.

I recommend its publication and believe that it will serve the organic electronics community, especially in the context of singlet fission, as a good benchmark for assessing impulsive vibrations.

Answer: We thank the Reviewer for suggesting to publish our paper as is and for acknowledging the significance of our work.

Reviewer #2 (Comments for the Author):

Souri et al present an ultrafast spectroscopy study of perfluoropentacene, focusing on the vibrational coherences generated through impulsive excitation. This is not a new technique, but advances in instrumentation have given it new currency and it is gaining renewed attention, including in the analysis of singlet fission (relevant to PFP here). One of the key aspects missing so far in the analysis of vibrational coherences is the correlation between the vibronic couplings that they report on and molecular packing. The authors use a creative approach here of growing large crystalline domains with the same packing but different orientations, on two different substrates, permitting VC analysis with polarization control that probes different coupling directions. The experimental work is of high quality, and the analysis is very well described. The study demonstrates the analytical power of this VC technique, especially when applied to crystalline materials, to probe the dynamics of vibronic coupling. It should be a significant interest to the ultrafast spectroscopy community and establishes a good model to follow, and I recommend publication. I raise a few minor issues below that would improve the manuscript.

Answer: We thank the Reviewer for the positive feedback and for recognizing the impact of our study.

1. The authors make reference to the UV-Vis spectra reported in reference 30. I would strongly encourage the authors to provide their own (ideally polarization-resolved) UV-Vis spectra; such a basic characterization is quite important in interpreting time-resolved spectroscopy results. This is even more the case when factoring in the pulse overlap parameters that impact vibrational coherence spectra. It is needlessly cumbersome for the reader to have to resort to a different publication to find the UV-Vis spectrum.

Answer: Following the advice of the Reviewer, we have recorded polarization-resolved UVVis spectra and X-ray diffraction data of the investigated samples. The data are briefly described in two new sections of the Supporting Information. X-ray diffraction data are shown as a new Fig. S1 and UV-Vis spectra as Fig. S2 and in Fig. R1 below.

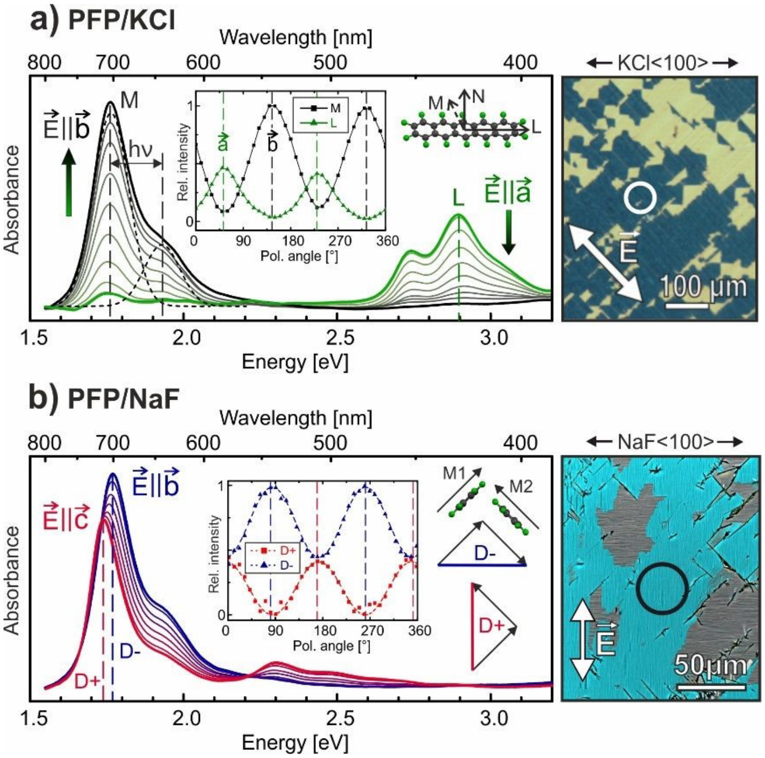

Fig. R1. Polarization-resolved UV/Vis absorption spectra of PFP films on a) KCl and b) NaF. The different molecular orientations give rise to optical anisotropies of the M- and L-polarized bands in (a). A vibronic side peak of the M band is shifted in energy from the main transition by $h\nu$, as depicted by dashed lines. For PFP on NaF (b), a Davydov-splitting between the bands polarized along $\vec{b}$ (D^{-}) and $\vec{c}$ (D^{+}) is marked by dashed lines (b). Polarized optical micrographs (right panels) show the different color appearances of single-crystalline domains within the films. Optical spectra have been recorded at the spots marked by open circles.

Changes: Two new sections to the Supporting Information have been added. The requested spectra are shown in Fig. S2. Additionally, we have updated the reference to this figure in the revised manuscript on page 7.

2. Page 25, line 35: '…suggest this is this out-of-plane…' is presumably missing an 'it'.

Answer: We apologize for the typo. We have corrected it.

Changes: The correction has been applied on page 23.

3. Based on the literature cited, there are different practices in the field for handling such vibrational coherence data; it would be useful for the authors to provide additional details about the data processing such as the use (or not) of window functions.

Answer: To analyze the vibrational coherence data, we have first subtracted the incoherent contributions from the pump-probe maps. We fit a multiexponential decay to the pump-probe transients for each detection energy (see Section 5 of the Supporting Information). This decay has

been subtracted from the pump-probe transients to give the residual map that is shown for PFP on NaF in Figure. 3a.

Fourier transform spectra have been calculated directly from the residuals for delay times of more than 120 fs, without applying any window function. This is now stated explicitly on p. 14 of the main manuscript.

Changes: On p.14, we now write: "For calculating the Fourier transform spectra, we used pump-probe transients for waiting times between 0.12 and recorded for up to 6 ps, except for the measurement performed on KCl with $\vec{a}$-axis excitation, where 3-ps scans were employed. Fourier transform spectra are calculated directly from the residuals, without applying any window functions." The information is also provided in the Supporting Information.

4. *There has been significant theoretical work on the interpretation of such vibrational coherences from Andrzejak and Petelenz (DOI: 10.1021/acs.jpcc.9b11772, DOI: 10.1021/acs.jpcc.0c06504 & DOI: 10.1021/acs.jpcc.4c01485). Among other things, these papers indicate that quantitative analysis of the vibrational coherences requires explicit consideration of the excitation pulse profile and its overlap with the absorption. Has this been factored into the FFT intensity analysis, or should it not matter given the pulse overlap used in these experiments?*

Answer: We thank the Reviewer for emphasizing the excellent work of Petelenz and coworkers on the role of coherent vibrational wavepacket dynamics for singlet fission in pentacenes. The Reviewer is right in noting that the pump profile can influence the intensity of vibrational modes, particularly in cases of narrow-band excitation. For this reason we used the spectrally very broad light pulses of a home-built noncollinear parametric amplifier in our study. Their spectrum, depicted in Fig. S3 of the Supporting Information covers the range from 1.5 eV (800 nm) to 2.05 eV (600 nm). Its spectral bandwidth exceeds the energy of highest frequency modes (< 200 meV) by far. The spectrum is designed to fully cover the lowest-lying absorption band of PFP, as shown in Fig. R1, including all relevant vibrational sidebands. In such a situation, the excitation pulse profile is essentially irrelevant for the Fourier transform analysis. This is confirmed by observing, in Fig. R2, that the spectral intensity of the pump laser is nearly flat in the region of large vibrational amplitude. To good approximation, we can therefore ignore the role of the pulse spectrum for the mode analysis.

Change: In the revised manuscript we now cite the work of Petelenz. We also emphasize that our laser spectrum is so broad that effects of the pump laser spectrum on the vibrational mode amplitude are weak and neglected in our analysis.

On p.7 we write: "The pulse spectrum fully overlaps the lowest-lying singlet exciton (X_{S1}) of the PFP crystals at about 1.8 eV and all relevant vibrational sidebands (see Fig. S2 in the Supporting Information)."

On p.17 we write: "Since the spectral width of our excitation pulses covers the entire absorption band of PFP, the laser profile does not significantly affect the mode amplitudes (cite Petelenz 2024)".

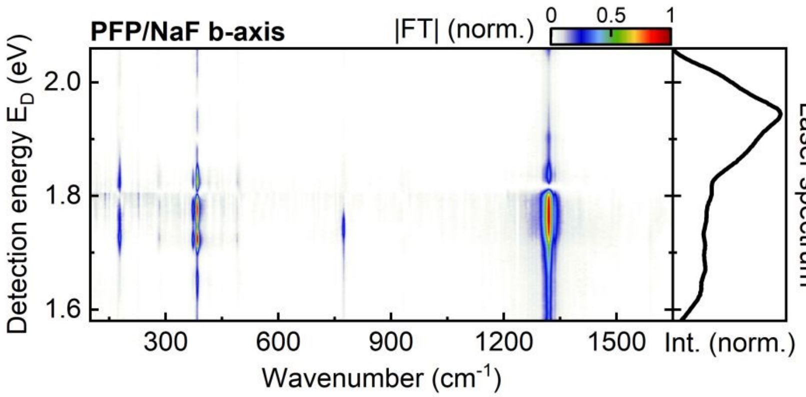

Fig. R2. Normalized energy-resolved Fourier transform spectra shown in Figure. 3a of the manuscript, together with the excitation pump profile.

Response to the Formatting Requests

1. Please provide full contact information for all authors in manuscript file: institution, city, state, postal code, country for each affiliation (states are required for United States addresses only). Postal codes are also required for addresses outside the U.S., for countries that have them. Each separate affiliation requires its own address information; they cannot be combined.

Answer: Full contact information is provided for all authors.

2. Please remove the section headings from your manuscript file, e.g., Introduction, Results and Discussion, Conclusion. Experimental section headings as well as paragraph headings are okay.

Answer: The section headings have been removed.

3. Please provide a brief, nonsentence description of the actual contents of each Supporting Information file.

Answer: A brief description of each section of Supporting Information file is provided.

4. Please include author names, article titles, journal name, publication year, and at least the first page number for each reference citation for the following incomplete journal references: 29.

Answer: Ref. 29 has been updated.
